# A Method for the Production of Recombinant VSVs with Confirmation of Biological Activity

**DOI:** 10.32607/actanaturae.27314

**Published:** 2024

**Authors:** V. D. Moroz, N. B. Gasanov, A. D. Egorov, A. S. Malogolovkin, M. O. Nagornykh, E. N. Subcheva, E. S. Kolosova, A. Yu. Fizikova, R. A. Ivanov, A. V. Karabelsky

**Affiliations:** Sirius University of Science and Technology, Krasnodar Region, Sirius, 354340 Russian Federation; First Moscow State Medical University (Sechenov University), Moscow, 119435 Russian Federation

**Keywords:** oncolytic viruses, vesicular stomatitis virus, cancer, melanoma, cytopathic effect, recombinant vesicular stomatitis virus

## Abstract

The design of new effective cancer treatment methods is a promising and important research field in translational medicine. Oncolytic viruses can induce immunogenic cell death by activating the body’s immune system to recognize tumor cells. This work presents the results for optimizing the production of recombinant vesicular stomatitis viruses (rVSVs). To ensure the assembly of viral particles, we developed the HEK293TN-T7 cell line, which stably expresses DNA-dependent RNA polymerase 7 for viral genome transcription, and obtained helper plasmids encoding viral genes under the control of the CAG promoter. The oncolytic activity of the purified virus preparation was assessed in a murine model of B16F10Red melanoma cells expressing a red fluorescent protein. The presented method makes it possible to obtain purified viral preparations with a high titer and oncolytic activity. The amplification of viral particles in a HEK293 suspension culture allows for rapid scalability. Therefore, the developed approach can be used to obtain other recombinant VSV-based oncolytic viruses for tumor immunotherapy.

## INTRODUCTION

Oncolytic viruses have long been considered as a potent antitumor drug. To date, the development of new oncolytic viruses is one of the priority areas of tumor immunotherapy. According to recent data, there are currently more than 200 clinical trials on the safety and effectiveness of oncolytic virus-based drugs [1, 2]. The RNA-containing vesicular stomatitis virus (VSV) effectively infects different human and animal cells while being non-pathogenic to humans. Low virulence, a rapid replication cycle in the cytoplasm (without integration into the genome), possibilities to obtain high virus titers when producing VSV in mammalian (BHK-21 and HEK293) cells and genetically modify viruses, as well as the absence of neutralizing antibodies in the human population, make VSV an ideal candidate for producing viral vaccines, transgene delivery vectors, and oncolytic viruses [3, 4, 5]. Assembly of such rVSVs as rVSV-dG-GFP involves transfection of BHK-21 cells with five plasmids and co-infection of cells with either the vaccinia virus (VV) or another virus expressing T7 DNA-dependent RNA polymerase (T7 RNA polymerase) [6]. When generating biotechnological products for further use as drugs, it is necessary to minimize the number of helper viruses, in particular the replication-competent ones, since the latter can cause viral contamination. In addition, the resulting drug preparation should not contain VV or other virus particles [7]. There are currently few works with a comprehensive and detailed description of all stages of the production, purification, concentration of VSV-based oncolytic viruses, and assessment of their functional activity for further studies. This paper presents a method for the production of purified replication-competent model rVSV encoding the green fluorescent protein (rVSV-dM51-GFP) without the use of a helper virus.

## EXPERIMENTAL

**Plasmids and genetic engineering**

The following commercial plasmids were used for cloning: pBS-N-FT-Kan (cat# EH1013, Kerafast, USA), pBS-P-FT-Amp (cat# EH1014, Kerafast), p B S - L - F T-A m p ( c a t # E H 1 0 1 5 , Ke r a f a s t ) , pBS-G-OT-Kan (cat# EH1016, Kerafast), pCAGGSG- Kan (cat# EH1017, Kerafast), pVSV-dG-GFP 2.6 (cat# EH1026, Kerafast), and pCAG-T7pol (cat# 59926, Addgene). *Escherichia coli *strains (DH5-alpha, NEB® Stable (cat# C3040H, NEB, Great Britain)) were used for plasmid amplification. Genetic transformation, hydrolysis by restriction endonucleases, ligation, gel electrophoresis, and DNA isolation were performed using standard protocols and the recommendations of the enzyme manufacturers [8]. Commercial reagent kits (cat. # BC021L and cat. # BC124, Evrogen, Russia) were used for DNA isolation. Targeted mutagenesis of the *VSVM *gene encoding the VSVM protein was performed by inverse PCR using pVSV-dG-GFP 2.6 (cat# EH1026, Kerafast) as a template and specific primers:

forward GACACCTATGATCCGAATCAATTAAGATATGAGA;

reverse CTCGTCAACTCCAAAATAGGATTTGTCAATTGGA.

*VSVG *f r o m t h e p C AG G S - G - K a n p l a s - mid was cloned into the mutagenesis plasmid pVSV-dG-dM51-GFP at NheI/XbaI sites to generate the pVSV-dM51-GFP plasmid, which is required for the assembly of replication-competent rVSV-dM51-GFP. To obtain helper plasmids with the CAG promotor (pCAG-VSVL, pCAG-VSVN, and pCAG-VSVP), *N *and *P *gene sequences were cloned at the EcoRI/NotI sites and the *L *gene was cloned at the XbaI/NotI sites in pCAG-T7pol. The genes were amplified using specific primers; the plasmids pBS-N-FT-Kan, pBS-P-FT-Amp, and pBS-L-FT-Amp were used as templates for the amplification of *VSVN*, *VSVP*, and *VSVL*, respectively. The correct assembly of vectors was verified by Sanger sequencing using an ABI 3500 Genetic Analyzer (Applied Biosystems, USA) in standard conditions with the reagents recommended by the manufacturer.

**Production of HEK293TN-T7 and BHK-21-T7 cells**

The T7-RNApol gene encoding the T7 RNA polymerase was cloned in the retroviral vector pBabe-bleo (Plasmid #176) at BamHI/SalI sites. The accuracy of the pBabe-bleo vector assembly was verified by restriction enzyme analysis and Sanger sequencing.

To obtain the retrovirus, the resulting plasmid was used for the transfection of Phoenix-AMPHO (CRL-3213 – ATCC) cells with Lipofectamine® 3000 (cat# L3000015, Thermo Fisher Scientific) according to the manufacturer’s instructions. HEK293TN and BHK-21 cells were infected with a cultural medium containing the obtained retrovirus for 3 h during each 12-h interval. Selection was performed within a week using 100 μg/ml of zeocin (cat# R25005, Invitrogen by Thermo Fisher Scientific). The surviving cells were seeded and screened 2 weeks after the start of selection based on the presence of a T7-RNApol insertion in the cell genome determined by PCR with specific primers. A clone of HEK293TN-T7 and BHK-21-T7 cells was selected for further studies. The activity of T7 RNA polymerase was confirmed in the clones by detecting luminescence using the Luciferase Assay System (Promega, USA). For this, the cells were transfected with the following expression vectors: plasmids pEGFPN3 (cat# 632515, Clontech), pSmart_5’-Mod-FFLuc-3’-Mod [9], pCAG-T7pol, and pSmart_5’-Mod-FFLuc-3’-Mod (positive control).

**Production and amplification of rVSV-dM51-GFP**

For rVSV-dM51-GFP assembly, 80–85% confluent HEK293TN-T7 cells in 12-well plates were transfected with pCAG-VSVN, pCAG-VSVP, pCAG-VSVL, pCAGGS-G, and pVSV-dM51-GFP at a 3:5:1:4:8 ratio, respectively, using PEI (a 5:1 ratio) and total DNA (10.5 μg). Cultural media, obtained 72 h after transfection, were used for further virus amplification in either adherent BHK-21 cells (cat# 85011433, ECACC General Collection) or suspension HEK293 cells in a serum-free medium (BalanCD HEK293, Irvine Scientific). Virus-containing supernatants (MOI = 10-4) obtained by centrifugation (3,000 *g*) of the culture medium from the previous stages of rVSV-dM51-GFP production were added, and the cells were incubated for 72 h. The culture medium was collected at all stages of virus production, centrifuged at 3,000 *g *and either stored at –80°C or used immediately for repeated virus transduction, isolation, purification, analysis, etc. After virus amplification in HEK293 cells, virus-containing supernatants were passed through 0.45-μm filters, concentrated (300 kDa, Vivaspin 6, VS0651, Sartorius) 100- to 200-fold and mixed with the standard phosphate-buffered saline (PBS, pH 7.4) for storage, purification, and analysis.

**Purification of rVSV-dM51-GFP by ultracentrifugation**

For p u r i f i c ation and concent r ation o f rVSV-dM51-GFP by ultracentrifugation (UCF), viral particles were purified and precipitated through a sucrose cushion (20% sucrose solution in HEN buffer: 10 mM HEPES pH 7.4, 1 mM EDTA, and 100 mM NaCl). The virus-containing supernatant was transferred into UCF tubes (cat# 344061, Beckman Coulter), and 4 ml of a 20% sucrose solution was added under the supernatant layer. The tubes were ultracentrifuged at 120,000 *g *and 4°C for 1 h, the supernatant was removed, and the precipitate was resuspended in 500 μl of an ET buffer (1 mM Tris-HCl, pH 7.5, 1 mM EDTA, and 10% DMSO) and incubated for 2 h at 4°C. The second UCF stage was performed in a discontinuous sucrose-density gradient with a HEN buffer with three different densities (25, 45, and 60%). Samples were centrifuged at 130,000 *g* and 4°C for 16 h. The band at the boundary between the 25% and 45% layers was extracted. At the third stage, the resulting sample was diluted 12-fold in standard PBS (pH 7.4). The samples were centrifuged at 120,000 *g *and 4°C for 1 h. The precipitate containing the purified viral fraction was dissolved in the required volume of PBS (pH 7.4).

**Analysis of rVSV-dM51-GFP samples by electron microscopy**

A Crossbeam 550 scanning electron microscope (Carl Zeiss, Germany) was used in the scanning transmission electron microscopy (STEM) mode. The sample was applied onto copper grids pretreated with air plasma for 10 s (formvar/carbon (200 mesh), cat#BZ31022a, EMCN, China) using a Zepto Plasma Cleaner (Diener Electronic). The samples were incubated for 2 min, and the grid was washed with double- distilled water and contrasted with 1% aqueous uranyl acetate (cat#0379, Polysciences Inc.) for 1 min. The resulting sample grids were dried in air at room temperature. The samples were visualized at an accelerating voltage of 30 kV.

**Analysis of rVSV-dM51-GFP samples**

The protein composition of the samples was analyzed by polyacrylamide gel electrophoresis (PAGE) under denaturing conditions using sodium dodecyl sulfate (SDS-PAGE) according to the standard protocol [10]. The rVSV-dM51-GFP preparation titer (TCID50/ml) was calculated using the Reed–Muench method [11].

**Production of B16F10red cells**

B16F10Red cells were obtained from B16F10 murine melanoma cells (third passage) from the Collection of Tumor Strains of the Federal State Budgetary Institution Blokhin National Medical Research Center of Oncology of the Russian Ministry of Health. B16F10 cells were obtained from murine C57BL/6 cells. B16F10 cells were transduced with viral particles from the culture medium of HEK293TN cells, previously transfected with the plasmids pMD2.G (cat# 12259, Addgene), pMDLg/pRRE (cat# 12251, Addgene), pRSV-REV (cat# 12253, Addgene), and a plasmid encoding the fusion protein H2B-Katushka2 under the EF1a promoter. Cells that survived in the selective medium were seeded in a 25 cm^2^ culture flask. Colonies with the highest fluorescence intensity of the H2B-Katushka2 reporter were selected. The fluorescence intensity in the resulting B16F10Red cell subline was detected using a microscope with a fluorescence filter (Carl Zeiss Axio Vert.A1).

**Monitoring of the B16F10Red cell number using lncuCyte S3**

To study the changes in the number of fluorescent B16F10Red cells, cells infected with viral particles with different rVSV-dM51-GFP dilutions were incubated for 84 h according to the manufacturer’s recommendations using a lncuCyte S3 Live Cell Analysis System. The number of fluorescent cells was counted every 2 h.

## RESULTS AND DISCUSSION

**Fig. 1:**
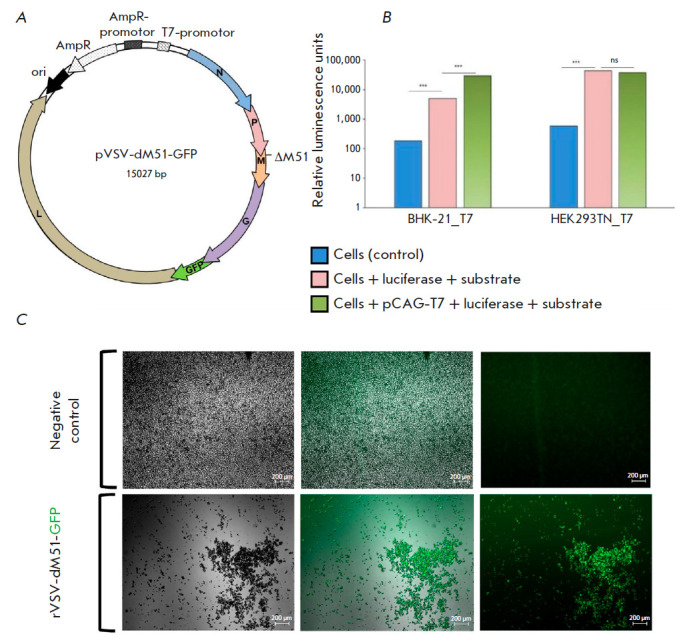
Assembly of rVSV-dM51-GFP from plasmids. (*A*) – Plasmid vector map for the assembly of replication-competent rVSV-dM51-GFP variants. The dM51 mutation and genes encoding VSV proteins are indicated, including the integrated VSV G gene. (*B*) – Luminescence of BHK21-T7 and HEK293TN-T7 cells. (*C*) – Micrographs of HEK293TN-T7 cells 72 h after transfection. The upper table presents the negative transfection control with the plasmids necessary for rVSV-dM51-GFP assembly from the plasmids in the absence of the vector encoding the viral genome (pCAG-VSVL, pCAG-VSVN, and pCAG-VSVP); the lower table shows the cells transfected with all plasmids (pCAG-VSVL, pCAG-VSVN, pCAG-VSVP, and pVSV-dM51-GFP) necessary for rVSV-dM51-GFP assembly

The conventional method for rVSV assembly [6] includes transfection of BHK-21 cells with five plasmids expressing individual genes and the complete VSV genome and co-infection of cells with either the vaccinia virus or another virus expressing T7 RNA polymerase, which is required for rVSV assembly [6]. The use of individual viruses expressing T7 RNA polymerase for rVSV assembly has a number of disadvantages, which are mentioned above. Co-transfection of cells with rVSV assembly plasmids and a plasmid encoding the T7 RNA polymerase gene makes it possible to avoid the unadvisable use of a helper virus for rVSV assembly. The use of an additional plasmid for transfection increases the DNA load on cells, which can cause cytotoxicity and affect virus assembly. For this reason, we obtained HEK293TN-T7 and BHK-21-T7 cells expressing T7 RNA polymerase. To assess the effectiveness of polymerase expression, we measured the luminescence of the cells transfected with a plasmid carrying the firefly luciferase gene under the T7 RNA polymerase promoter. Luciferase expression under the T7 RNA polymerase promoter in HEK293TN-T7 cells reaches a level similar to that in the positive control (cells transfected with a plasmid carrying the T7 RNA polymerase gene). However, at the same time, the luciferase expression level exceeds that in BHK-21-T7 cells (*Fig. 1B*). Therefore, we used HEK293TN-T7 cells in further studies for the assembly of model rVSV particles. The pVSV-dM51-GFP plasmid with a M51 deletion in VSV M (dM51) was used as a model virus to develop an optimized technique for obtaining purified rVSV preparations. It is known that rVSV with this deletion does not suppress interferon expression upon its entry in live cells, which makes this modification valuable in terms of the safety of rVSV-based drugs [12]. The model virus genome also contains *VSVG*, which encodes the envelope glycoprotein required for virus entry into the cell. This makes the virus replication-competent; in addition, it also makes it possible to avoid pre-transfection of cells with a *VSVG*-encoding plasmid for the production of virus preparations [6, 13]. To introduce modifications into the model virus genome, we obtained the pVSV-dM51-GFP plasmid carrying the dM51 deletion and *VSVG* (*Fig. 1A*). The plasmid was used for co-transfection with helper plasmids (pCAG-VSVL, pCAG-VSVN, and pCAG-VSVP) at the stage of virus assembly. To increase the effectiveness of model rVSV-dM51-GFP assembly, we also constructed helper plasmids with the CAG promoter based on the data on promoter use for rVSV assembly [14] and evidence of protein synthesis enhancement by the CAG promoter in HEK293F cells [15]. BHK-21 and Vero cells are usually used for rVSV production [16]. However, we observed efficient rVSV-dM51-GFP assembly only in HEK293TN-T7 cells (*Fig. 1C*). Neither the GFP nor CPE signal was observed in BHK-21-T7 cells, or even in BHK-21 and Vero-76 cells transfected with the additional plasmid pCAG-T7pol.

**Fig. 2:**
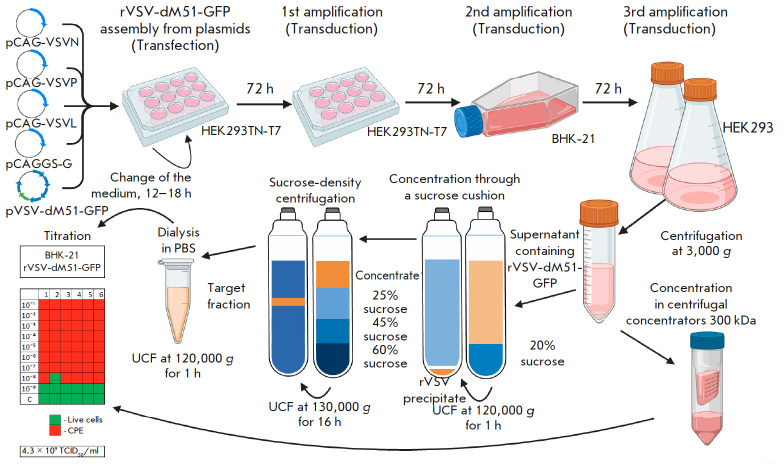
Schematic representation of the protocol for preparation of purified rVSV-dM51-GFP in adherent and suspension cell cultures

**Fig. 3:**
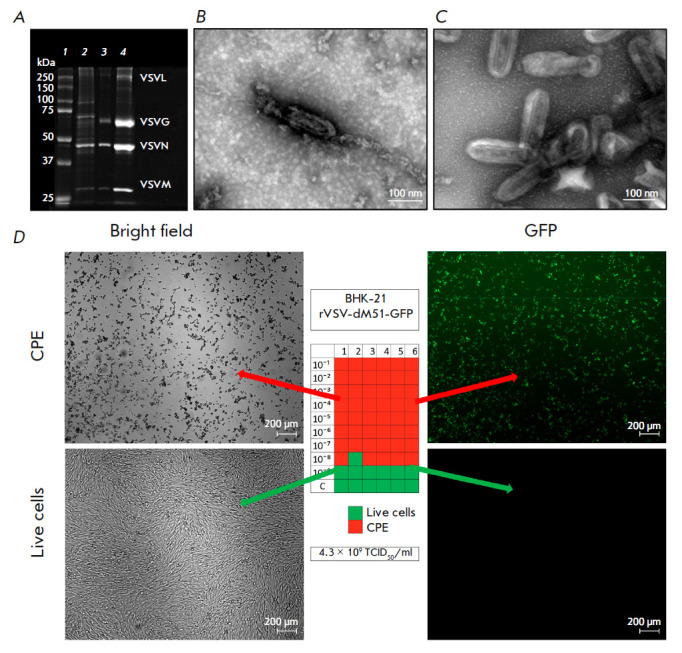
Analysis of rVSV-dM51- GFP samples. (*A*) – SDS-PAGE (from left to right):* 1 *– protein molecular weight marker (10–250 kDa),* 2 *– rVSV-dM51-GFP supernatant after CF at 3,000 *g*,* 3 *– rVSV-dM51-GFP fraction after the second UCF stage,* 4 *– purified rVSV-dM51-GFP after the third UCF stage; (*B*) – STEM micrograph image of rVSV-dM51-GFP before UCF purification (magnification, × 130,000). (*C*) – STEM micrograph of rVSV-dM51-GFP after UCF purification (magnification, × 130,000). (*D*) – TCID50/ml in BHK-21 cells and the micrographs of BHK-21 cells in the presence (top) and absence (bottom) of CPE

**Fig. 4:**
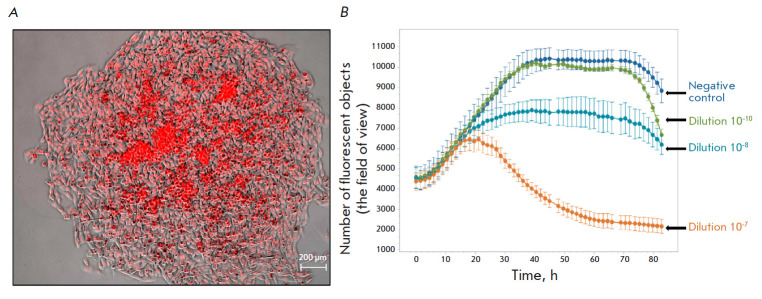
The dose–response relationship for rVSV-dM51-GFP in B16F10Red cells. (*A*) – micrographs of B16F10Red cells; (*B*) – CPE in B16F10Red cells after addition of rVSV-dM51-GFP at different dilutions

The ratio of plasmids used for HEK293TN-T7 transfection resulting in efficient rVSV-dM51-GFP assembly differs from the ones used in previously published protocols [6, 14]. At the same time, the plasmid ratios used in the study by Whitt M. [6] generated GFP and CPE signals similar to those in the negative control (data not shown, since they coincided with the negative control, as presented in *Fig. 1C*). We assume that the optimal plasmid ratios for the assembly of model rVSV-dM51-GFP particles vary under different conditions. Hence, when rVSV is not assembled, the plasmid ratio can be adjusted and the optimal ratio can be determined for the given conditions. Due to the lack of comprehensive protocols for rVSV production, we developed a method with in-detail description of all stages of the production of a purified preparation of the model rVSV virus, including titer determination and oncolytic activity evaluation (*Fig. 2*–*4*).

Production of the rVSV preparation in a cell suspension culture greatly facilitates the scaling up of the technological process and simplifies the scaling of laboratory technology to the use of industrial bioreactors and, therefore, the industrial production of rVSV batches [17]. The use of a serum-free medium for cultivation, e.g. a HEK293 suspension culture, eliminates the need to test the drug for the presence of components of animal origin [18]. Optimization of the HEK293 cultivation process, for instance, by using culture feeds, can significantly increase the virus titer [17]. To purify the rVSV-dM51-GFP preparation of contaminants and inhibitory particles and concentrate the preparation, we performed a three-step purification of samples by sucrose-density UCF (*Fig. 2*). The purification steps included concentration of viral particles and purification in a sucrose gradient, followed by isolation and dialysis of the target viral fraction in PBS (pH 7.4). In similar protocols for VSV purification, the order of the stages may vary; for example, the stage of concentration through the so-called sucrose cushion may be the final step [19] or even absent altogether [20]. In our study, we centrifuged the culture medium containing rVSV-dM51-GFP through 20% sucrose not only to concentrate the sample, but also to partially pre-purify it from contaminants and thereby increase the efficiency of the following second stage of purification in a sucrose gradient. The following sucrose concentrations were used at the second UFC stage: 25%, 45%, and 60% [21]. The final purification step included dialysis in PBS (pH 7.4) using re-precipitation by UCF (*Fig. 2*). UCF can lead to loss and damage to viral particles and, as a consequence, a decrease in the viral preparation titer. To verify the titer and purity of the rVSV-dM51-GFP preparations obtained by using the proposed protocol (*Fig. 2*), we analyzed the samples by SDS-PAGE, TCID50, and STEM (*Fig. 3*). A change in the intensity of the SDS-PAGE bands corresponding to five rVSV proteins [22, 23] and the absence of non-specific bands indicate an increase in the viral concentration at each purification stage (*Fig. 3A*).

A STEM analysis of the viral samples also showed that UCF of the rVSV-dM51-GFP preparation resulted in an increase in the number of viral particles in the field of view and a decrease in the number of contaminants (*Fig. 3 B,C*). To confirm the viability of the viral particles, we measured rVSV-dM51-GFP titers before and after UCF by using TCID50 as a method that determines the number of infectious viral particles causing a cytopathic effect [11]. In addition to the SDS-PAGE and STEM data, the rVSV-dM51-GFP titer in the supernatants before the concentration stage (2 × 10^8^ TCID50/ml) was lower than that after viral purification and concentration by UCF (4.3 × 10^9^ TCID50/ml) (*Fig. 3D*). In addition to performing qualitative and quantitative analyses, we also studied the oncolytic properties and dose– response relationship of the model rVSV preparation obtained using the proposed approach in B16F10 murine melanoma cells (*Fig. 4B*). These cells are often used to evaluate the therapeutic properties of various drugs, including oncolytic viruses, and, in particular, in *in vivo *studies [24, 25, 26]. Visualization of living cells using fluorescent proteins makes it possible to monitor cancer cell growth both *in vitro* and *in vivo*. This, in turn, allows for an evaluation of the therapeutic effect of anticancer drugs. To assess the dose–response relationship *in vitro*, we obtained RFP-expressing B16F10 cells (B16F10Red) (*Fig. 4A*) and conducted intravital cell number monitoring on lncuCyte S3 (*Fig. 4B*). A similar growth trend was observed in virus-free variants and variants containing high dilutions of the viral preparation (**108 and 1010**): the growth (~0–40 h), plateau (~40–75 h), and cell death phases (≥~75 h). In case of using the lowest dilution of the viral preparation (**107**), the cell growth pattern differed significantly: the cell death phase was observed approximately 25 h after the growth phase. We plotted the curves of the number of fluorescent objects on the viral preparation dilution and observed the dose–response relationship between the viral amount and the number of living cells (*Fig. 4B*). Based on those results, we conclude that the model virus particles do not lose their viability and oncolytic properties while they retain the ability to lyse cancer cells at high viral dilutions (**107**–**108**) after the abovementioned stages of virus assembly, amplification, and purification.

## CONCLUSION

We have developed a scalable method for producing purified preparations of model rVSV-dM51-GFP without the use of a helper virus. The protocol includes the stages of production, purification, titer determination, and analysis of the virus oncolytic activity. The model virus preparation obtained by the above-described approach can be used to assess its therapeutic characteristic in *in vivo *syngeneic murine models with B16F10 cells and compare it with enhanced rVSV variants with immunostimulatory characteristics [12, 26, 27].

## Abbreviations

VSV: vesicular stomatitis virus
rVSV: recombinant vesicular stomatitis virus
GFP: green fluorescent protein
CPE: cytopathic effect
MOI: multiplicity of infection
CF: centrifugation
UCF: ultracentrifugation
TCID50/ml: tissue culture infectious dose of the virus
bp: base pairs
RFP: red fluorescent protein
